# A case report of renal calyceal diverticulum with hypertension in children and review of literature

**DOI:** 10.1186/s12887-021-03081-5

**Published:** 2022-01-11

**Authors:** Yongxiang Zhao, Ruimin Zhang, Ye Yun, Xiangming Wu, Haowei Li, Jun Wang, Wei Wang, Chunmei Jia, Hongcheng Song

**Affiliations:** 1 The Fourth Hospital of Baotou, Baotou, Inner Mongolia China; 2 Beijing Children’s Hospital, Beijing, China

**Keywords:** Children, Calyceal diverticulum, Renal cyst, Hypertension

## Abstract

**Background:**

Renal calyx diverticulum refers to a cystic lesion covered with the transitional epithelium in the renal parenchyma. Although there is no clear evidence that calyx diverticulum can cause hypertension, there exists a close association between the two, and there are few related reports. Herein, we reported the case of a child with renal calyx diverticulum complicated with hypertension and summarized the diagnosis and treatment.

**Case presentation:**

Physical examination of the patient, an 11-year-old child, revealed a left renal cyst with hypertension (155/116 mmHg). There were no related symptoms. Routine urine and blood biochemical examinations showed no abnormalities. Imaging revealed left renal cyst compression causing the hypertension. She underwent renal cyst fluid aspiration and injection of a sclerosing agent into the capsule, but her blood pressure increased again 3 days postoperatively. Color Doppler ultrasonography showed that the size of the left renal cyst was the same as that preoperatively. To further confirm the diagnosis, cystoscopic retrograde ureteropyelography was performed to confirm the diagnosis of renal calyx diverticulum. Subsequently, renal calyceal diverticulum resection and calyx neck enlargement were performed. The operation went smoothly and the blood pressure returned to normal postoperatively. No abnormalities were noted at the 7-month postoperative follow-up.

**Conclusion:**

There exists an association between renal calyx diverticulum and hypertension. Therefore, hypertension can be considered a surgical indication for renal calyx diverticulum. Moreover, renal calyceal diverticulum in children can be easily misdiagnosed as a renal cyst. Therefore, it is important to be vigilant to prevent a series of complications, such as postoperative urine leakage, in such cases.

## Background

Renal calyceal diverticulum refers to a cystic lesion covered with the transitional epithelium in the renal parenchyma [1], which is connected to the calyceal or renal pelvis through a narrow passage. It is difficult to differentiate between this lesion, the renal pelvis, and paracal cystic diseases [2], which could easily lead to misdiagnosis. Presently, reports on hypertension caused by renal calyceal diverticulum in children are rare. Here we report a case of renal calyceal diverticulum with hypertension in The Fourth Hospital of Baotou.

## Case presentation

This study was approved by the ethics committee of The Fourth Hospital of Baotou (ethics approval number is not applicable for our ethics committee). Written informed consent was obtained from the patient’s parents.

**Fig. 1:**
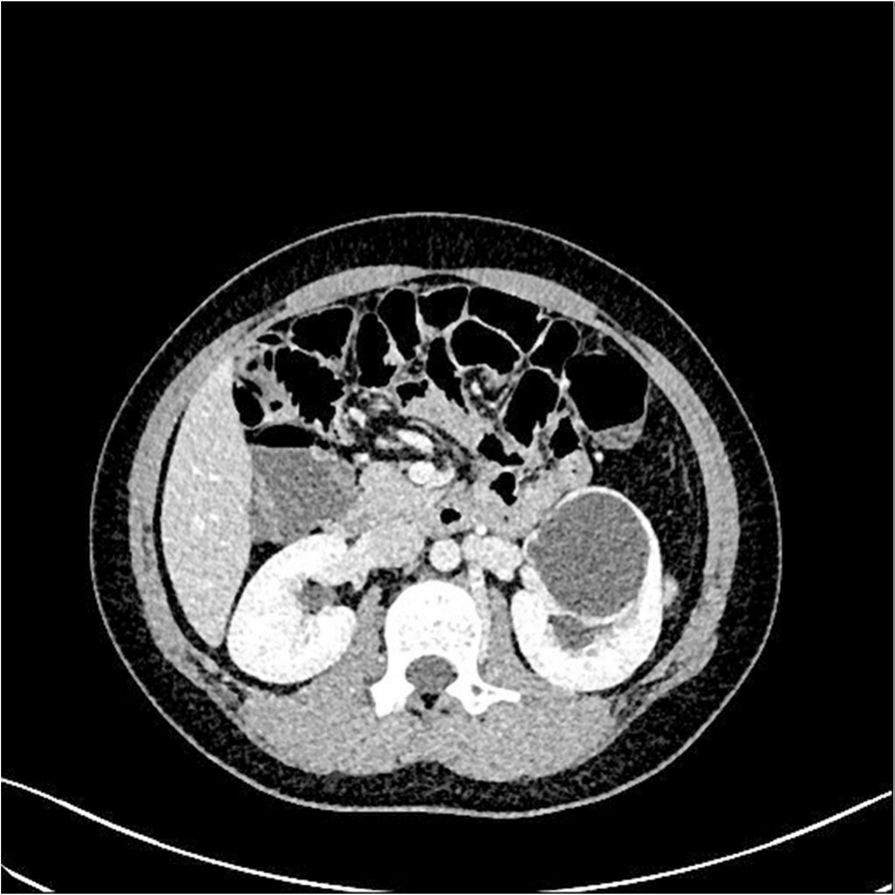
Preoperative urological CT. A rounded hypointense shadow (approximately 47.2 × 46.1 × 59.3 mm) with clear borders observed in the left kidney

**Fig. 2:**
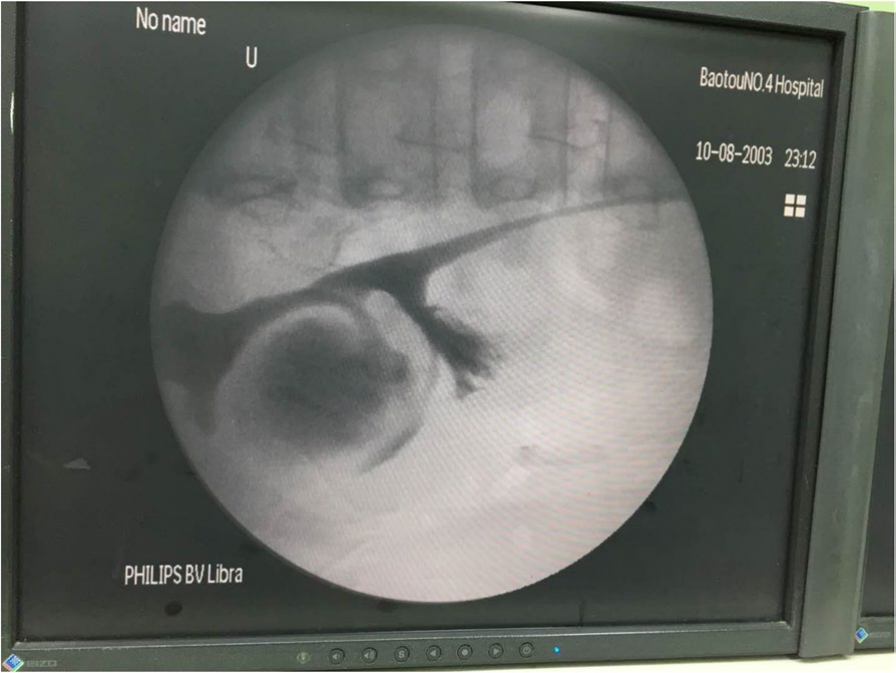
Intraoperative C-arm view. The left superior and inferior calyces were normal, the middle calyces were spherically dilated, and the diverticulum was attached to the renal pelvis

**Fig. 3:**
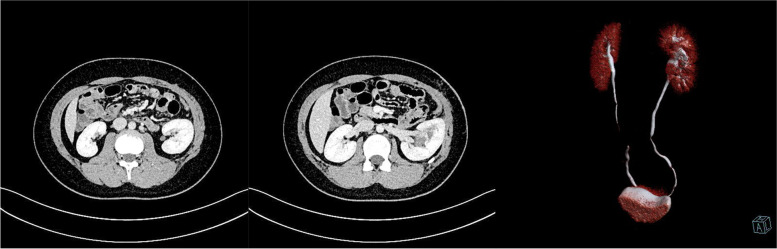
Seven-month postoperative urological CT. The left kidney was slightly larger, with irregular morphology of the superior parenchyma, slightly enlarged local renal pelvis, and normal renal structure, considering postoperative changes

The patient was an 11-year-old girl whose physical examination revealed left renal cyst with hypertension (blood pressure, 155/116 mmHg). She had no back pain and no other symptoms such as frequent, acute, or painful urination. Her blood pressure upon admission was 153/113 mmHg. Oral amlodipine besylate (benzenesulfonic acid amlodipine) was used to normalize the blood pressure, following which routine urine and blood biochemical tests were performed. Urological CT revealed normal size, shape, and location of both kidneys. The left kidney was round, had a clear outline, had low density, and measured about 47.2 × 46.1 × 59.3 mm in size, with clear boundaries on CT at approximately 6 HU. No significant enhancement was noted in enhanced CT for any of the phases. There was also no abnormal density in the fatty capsule surrounding both kidneys, no separation of the bilateral assembly system, and no thickening sign in the perirenal fascia (Fig. 1). Upon CTA of both renal arteries, both arteries were found to be of normal shape at their origins. There was no obvious stenosis and expansion. The left renal vein was normal and there was no obvious compression stenosis. Serum renin, serum angiotensin, serum aldosterone, thyroid function, plasma cortisol, and blood catecholamine levels were normal. Hypertension due to compression of the renal cyst was considered on the basis of imaging. She was admitted to the hospital and was monitored using color ultrasonography. Renal cyst fluid aspiration was performed, followed by intracapsular injection of a sclerosing agent. Her blood pressure returned to normal, but increased again on the third day. Repeat ultrasonography showed that the left renal cyst had returned to its pre-aspiration size. Hence, we noted the following: 1. The injection of a sclerosing agent in the renal cyst is ineffective in children; soon after aspiration, the cyst fills again; hence, it is important to be vigilant for renal calyx diverticulum. 2. Hypertension in children is associated with cysts. Cystoscopic retrograde ureteropyelography was performed to further clarify the diagnosis. After anesthesia, cystoscopic retrograde intubation of the left ureter showed normal left upper and lower renal calyces and a spherically dilated middle calyceal contrast entry with a cyst attached to the renal pelvis (Fig. 2). The diagnosis of diverticulum of the renal calyces was confirmed. Open resection of the calyx diverticulum and enlargement of the calyx neck were performed. Upon intraoperative incision of the thin diverticular wall of the renal calyces, the diverticulum was observed to originate from the middle calyces of the kidney and the neck of the calyces was apparently narrowed. The narrowed calyces were enlarged to a diameter of approximately 1 cm by probing, and the incision was sutured continuously with locked edges to prevent restenosis. Subsequently, most of the dilated diverticular wall was excised and the thicker residual part of the calyx wall was closed. The 8-Fr catheter was left in the calyx neck as a stent tube, and the operation went smoothly. The patient’s blood pressure decreased to normal on the postoperative day without the use of oral antihypertensive drugs, and the stent tube was removed 1 week postoperatively. At the 7-month postoperative follow-up and urological CT (Fig. 3), the patient showed good recovery, with no blood pressure abnormalities since discharge.

### Literature review

The PubMed database was searched using the search term “calyceal diverticulum” for articles published between January 2010 and December 2020. A total of 36 patients with calyceal diverticulum were reported in the literature, which, combined with this report, makes 37 cases. The average age of the patients was 21.2 years (1–82 years), and the average age of the children was 8.5 years (1–17 years) in 21 cases. There were four cases with no symptoms. No cases of hypertension in combination with renal diverticulum were reported. Fifteen cases were treated surgically, as described in Table 1 [3–20]. Two of these cases could not be treated owing to the severity of the disease.

**Table 1:** General information of patients with renal calyx diverticulum

No	Author (Country)	Year	Age	Sex	Diagnosis method	Diagnosis	Symptoms	Diverticulum size (mm)	Complications	Treatment modality	Prognosis
1	China Zhang Z et al. [3]	2018	38	Male	CT + pyelogram	Calvarial diverticulum	No urinary frequency, urinary urgency, hematuria, back pain, abdominal pain	66*50	None	Holmium laser percutaneous nephrological treatment of calcaneal diverticulum + dilatation at calcaneal stenosis	A CT scan of the kidney six months later showed a smaller cyst than before surgery, with no complications at follow-up
2	China Zhang Z et al. [3]	2018	23	Female	CT + pyelogram	Renal calyces diverticulum Glomerular diverticulum	Left renal colic with nausea and vomiting	65*52	None	Holmium laser percutaneous nephrological treatment of calcaneal diverticulum + dilatation at calcaneal stenosis	A CT scan of the kidney six months later showed a smaller cyst than before surgery, with no complications at follow-up
3	Morocco Smyth N, et al. [4]	2019	82	Male	Ureteroscopy	Diverticulum of renal calyces combined with stones	Mild intermittent right rib pain	28	None	Allopathic treatment + extracorporeal shock wave lithotripsy	No complications
4	Japan Mitome T, et al. [5]	2018	73	Female	CT + Ureteroscopy	Calvarial diverticulum	Asymptomatic hematuria	58	None	Laparoscopic radical nephroureterectomy (percutaneous nephroscopy further revealed a papillary lesion on the surface of the diverticulum, confirmed by pathological evaluation as squamous cell carcinoma.)	A CT imaging performed 2 months after this procedure confirmed recurrence and the patient received adjuvant systemic chemotherapy with cisplatin and gemcitabine. The patient went into septic shock during the first chemotherapy treatment. During chemotherapy, systemic therapy needed to be stopped and her systemic condition continued to deteriorate thereafter. At this point, the patient opted for palliative care only and died 4 months after radical nephroureterectomy.
5	Japan Yamasaki T, et al. [6]	2018	45	Female	Ureteroscopy	Ureteral stone secondary to ruptured right renal calyx diverticulum exudate	Severe pain in the right kidney	None	Pain and fever in the right hip	After ureteroscopy (ruptured exudate of diverticulum after ureteroscopy), postoperative treatment with urine culture plus antibiotics is done.	Perirenal extravasation on computed tomography scan at 3 months postoperatively
6	China Zhang R, et al. [7]	2015	51	Male	Ultrasound of the abdomen+ CT	Calvarial diverticulum	Left abdominal pain for 10 days, no hematuria or lower urinary tract symptoms	None	None	Partial left nephrectomy (due to chronic pain. Recurrent urinary tract infection, severe hematuria)	Not mentioned in the literature
7	China Peng YH, et al. [8]	2011	60	Male	Combined retrograde urography + CT + MRI urography	Diverticulum of renal calyces combined with left hydronephrosis and bilateral renal cysts	Low Back Pain	left kidney:101*81 73*61 right kidney:61*48 22*19	None	Patients receive non-surgical treatment	At 2 years of follow-up, the patient had no worsening of ipsilateral abdominal pain and no enlargement of the renal calyx diverticulum.
8	Spain Bonastre C, et al. [9]	2016	24	Female	Ultrasound of the abdomen+ CT	Calvarial diverticulum	Left back pain and fever	None	None	Laparoscopic nephrological diverticulectomy	Jackson-Pratt drainage tube was placed. CT on the third day showed that the diverticulum had subsided with no signs of recurrence.
9	India Sripathi V, et al. [10]	2017	10	Male	Ultrasound + CT	Type 2 renal calyx diverticulum	Swollen and palpable right kidney without fever or difficulty urinating	Bigger: 40*39 Smaller: 28*16	None	Robot-assisted laparoscopic suturing of diverticular neck	Still asymptomatic after 18 months.
10	China Pan Y, et al. [11]	2020	69	Female	CT + pyelogram	Bilateral bilateral bilateral renal insufficiency combined with left ureteral cyst and renal calyx diverticulum stone	Pain in the left lower back	Not marked with size	None	Holmium laser resection of ureteral cyst and holmium laser endoscopy of diverticular neck stricture revealed clear stones, which were removed via ureteral soft-scope holmium laser and nitinol basket. Double J ureteral stent was placed for 1 month	The ureteral stent was removed after 1 month and the patient was stone free on CT scan.
11	Denmark Pareek A, et al. [12]	2014	72	Male	CT	Renal tubular diverticulum	Pet-ct Discovery	Not marked with size	None	Severe disease inoperable	Died of heart disease
12	China Ng WM. et al. [13]		44	Female	X-Ray	Diverticular atresia and stone in the right renal calyx	Physical Examination Findings	Not marked with size	None	Holmium laser lithotripsy by RIRS + widening of the atretic wall (diverticular neck) + double J-tube placement	Follow-up KUBs at 2 and 4 weeks showed that the right renal stone was no longer visible
13	Canada Alwaal A, et al. [14]	2012	56	Female	CT + pyelogram	Calvarial diverticulum	Pain in the low back with no significant past medical history	20*21	None	Holmium laser percutaneous electrocautery for renal calyx diverticulum + double J-tube stenting	IVP was performed at 2, 11 and 24 months postoperatively, showing the disappearance of stones and a significant reduction in the size of the renal calyx diverticulum, which remained asymptomatic after 30 months.
14	Canada Alwaal A, et al. [14]	2011	64	Female	CT + pyelogram	Calvarial diverticulum	Low back pain and urinary tract infection	24*14	None	Holmium laser percutaneous electrocautery for renal calyx diverticulum + nephrostomy	An intravenous injection 12 months after surgery showed a significant reduction in renal calyx diverticula with no signs of stones. 30 months later it was still asymptomatic.
15	Germany Oh MM, et al. [15]		24	Female	CT	Bacterial sepsis after extracorporeal shock wave lithotripsy for renal calyx diverticulum stones	Low back pain, fever, general weakness	Size unknown	None	Emergency percutaneous nephrostomy with drainage + stone removal and diverticulectomy (open surgery)	No residual calyx diverticulum on follow-up intravenous pyelogram
16	Japan Nakano T, et al. [16]	2013	70	Male	CT + pyelogram	Infiltrative uroepithelial carcinoma of the diverticulum of the renal calyx with renal calculi	Health Screening Findings	80*50*45	None	Left laparoscopic radical nephrectomy (retroperitoneal approach)	No recurrence was observed during the 12-month follow-up period
17	America Ferroni MC, et al. [17]	2015	5	Female	pyelogram	Diverticulum of the renal calyces with large extrarenal dilatation	Daytime enuresis and frequent urination	82*42* 41	None	Robotic-assisted laparoscopic extra-renal partial resection of left calyx diverticulum	At the 2-week postoperative review, the patient had no concerns and denied any residual pain. The ureteral stent was removed 4 weeks postoperatively without complications.
18	Poland Przemysław Bombiński1 [18]	2015	5.5	Female	CT+ pyelogram	Calyceal diverticula	Lumbar spine pain, fever up to 40 degrees, poor response to antipyretics	23	None	Patients receive non-surgical treatment	No significant change in diverticulum at 2 years of follow-up
19	China Chun-Chen Lin [19]	2015	9	Male	CTU + Tc-99 m DTPA renal scan	Calyceal diverticula with stones	Abdominal pain	20	Stone	None	None
20	China Chun-Chen Lin	2015	9	Male	CTU + Tc-99 m DTPA renal scan	Calyceal diverticula	Rt flank pain	24	None	None	None
21	China Chun-Chen Lin	2015	5	Male	CTU + Tc-99 m DTPA renal scan	Calyceal diverticula	Bronchopneumonia with abdominal pain	23	None	None	None
22	China Chun-Chen Lin	2015	10	Female	CTU + Tc-99 m DTPA renal scan	Calyceal diverticula	Intermittent abdominal pain, Rt flank knocking pain	19	None	None	None
23	China Chun-Chen Lin	2015	15	Female	CTU + Tc-99 m DTPA renal scan	Calyceal diverticula	Lt flank pain	23	None	None	None
24	China Chun-Chen Lin	2015	3	Female	CTU + Tc-99 m DTPA renal scan	Calyceal diverticula	Nephrotic syndrome	17	None	None	None
25	China Chun-Chen Lin	2015	7	Female	CTU + Tc-99 m DTPA renal scan	Calyceal diverticula	Rt flank pain	40	None	None	None
26	China Chun-Chen Lin	2015	3	Female	CTU + Tc-99 m DTPA renal scan	Calyceal diverticula with stones	Fever with pyuriay	26	Stone	None	None
27	China Chun-Chen Lin	2015	9	Female	CTU + Tc-99 m DTPA renal scan	Calyceal diverticula	Precocious puberty	12	None	None	None
28	Turkey Demet Alaygut [20]		114. 6 ± 68.4 (12–204) month	5 F/4 M	MRU	Calyceal diverticula	2 urolithiasis, 3 urinary tract infection	20.44 ± 6.4 mm(10–30)	None	None	51.6 ± 22 (23–90) month No complications

## Discussion and conclusion

The prevalence of hypertension in children in China is 14.5% and is higher in males (16.1%) than in females (12.9%) [21]. Hypertension in early childhood often has no obvious symptoms. Common causes of this condition include congenital aortic stenosis, congenital renal hypoplasia, congenital urinary tract malformations, renal artery stenosis, latent glomerulonephritis, and adrenal disease. There are very few reports stating that hypertension in childhood is caused by renal calyceal diverticulum. Diverticula of the renal calyces in children is clinically rare, with a documented incidence of 0.6% [22]. A renal calyceal diverticulum is a sac-like structure that is located in the renal parenchyma and connected to the renal calyx. According to the different connection positions of the passage, it can be divided into type I and type II diverticulum. The former is connected to the minor renal calyx, mostly at one pole of the kidney, and the latter is connected to the major renal calyx, mostly at the central part of the kidney [23].

Secondary stones are more common in cases of calvarial diverticula, with a reported clinical incidence of 9.5–50.0% among cases of calvarial diverticula caused by outflow tract obstruction and urinary reflux [24]. In the last decade, 10 of 36 cases of renal calyx diverticulum were complicated by stones. Further, among other clinical manifestations, pain was the most common symptom (17/36), and concomitant symptoms, such as fever (8/36) or bladder irritation, were often present when secondary urinary tract infection was present. In children, abdominal pain was the most common symptom (6/21). Four children had a combination of stones, and the diverticulum was rarely diagnosed in children. Considering that it resembles other cystic lesions of the kidney, further evaluation of children with renal cysts should be done in the presence of back pain, recurrent urinary tract infections, hematuria, and stones [20]. Per our experience, this disease may be misdiagnosed as renal cysts using ultrasonography and plain or even enhanced CT. Therefore, delayed contrast or delayed enhanced CT should be performed when the diagnosis is not confirmed or when renal calyx diverticulum is suspected. In addition, retrograde urography may also be performed to clarify the diagnosis. This CT + pyelography approach was used to confirm the diagnosis in 16 of 36 cases of renal calyx diverticulum. In cases for which imaging is not possible, cyst fluid aspiration can be performed under ultrasound-guided localization to assist in the diagnosis based on the cyst fluid composition [3], which has not been reported in the literature in the last decade.

Renal calyceal diverticulum is not common among children, and only 20% of the cases eventually present symptoms [10]. In the last decade, 36 cases of renal calyx diverticulum were investigated, among which 21 were of children. Further, 15 patients were treated surgically for more obvious complications and 2 were treated symptomatically because they could not tolerate surgery. The treatment of renal calyx diverticulum needs to be determined by clinical symptoms. Symptomatic treatment can be given to children with no symptoms or mild clinical symptoms and for small-sized diverticula. Regular follow-up and surgery are recommended for children with large diverticula (> 4 cm) or complications [1]. Kavukcu et al. [25] proposed that the treatment of the diverticulum depends on the complications, including repeated kidney infections, hematuria, and symptomatic kidney stones. Considering our experience, we believe that for children with hypertension and renal calyceal diverticulum, if the cause of hypertension cannot be determined, surgery should be performed in time. In our case, retrograde ureterography was performed preoperatively and a ureteral stent tube was left in place as a marker. The wall of the diverticulum was incised, the stenotic opening was found, the opening of the diverticulum was enlarged, the ureteral stent tube was visible and was determined to be connected to the renal pelvis, and the enlarged diverticulum opening was sutured with locked edges, with the stent tube left in place for support. The diverticulum wall was excised and the portion with the remaining renal tissue was sutured closed. The stent tube was removed 1 week postoperatively, and the patient’s blood pressure returned to normal. The diverticulum did not recur on repeat ultrasonography and enhanced CT performed 7 months postoperatively, and the patient’s blood pressure continued to remain normal.

Long et al. [26] suggested that ureteroscopy should be selected for middle and upper renal calyceal diverticula, whereas laparoscopic treatment should be considered first for larger exogenous lesions and lower pole diverticula. In well-equipped hospitals, robot-assisted technology can be used to help accurately identify and ligate the opening of the renal calyceal diverticulum to achieve better surgical outcomes [10, 27]. If diverticulum-induced stones are found in the preoperative examination, endoscopic percutaneous nephrolithotomy or laparoscopic treatment can be performed according to the location. However, ESWL is not ideal in the treatment of such stones [28, 29].

The diagnosis in this case was renal calyceal diverticulum with hypertension. Although there is no clear evidence stating that renal calyceal diverticulum can cause hypertension, there exists a close association between the two. During kidney transplantation, high blood pressure can also be “transplanted” along with the kidney, which sufficiently proves the important role of the kidney in blood pressure regulation. Any kidney disease could cause abnormal blood pressure [30–32]. Presently, the mechanisms by which kidney diseases can increase blood pressure mainly include sodium ion retention, renin–angiotensin system (RAS) dysregulation, sympathetic nervous system dysfunction, and endothelial cell-mediated vasodilation impairment [31]. In recent years, some scholars have discovered that the renal enzyme system is closely related to blood pressure [33], and its way of regulating blood pressure might be related to the metabolism of NADH [34] and catecholamines and the transport of sodium in the proximal renal tubules [35]. Malyszko et al. [36] pointed out that the mechanism underlying blood pressure regulation by renal enzymes and the relationship with dopamine receptors and endothelial function need further confirmatory evidence, and the specific mode of action is still unclear. Our patient had type II renal calyceal diverticulum with a large cyst. The cause of hypertension in this case could not be determined preoperatively, and the blood pressure immediately returned to normal postoperatively. Therefore, we considered the increase in blood pressure to be caused by cyst compression. The major reasons for this could be as follows: (1) Expansion of the cyst connected with the renal calyx compressed the sympathetic nerve branch, thereby causing constriction of the afferent arteriole of the corresponding nephron. (2) Compression caused renal ischemia and RAS activation. The formation of bladder cysts and long-term compression of the kidney might result in the loss of some nephrons and affect blood pressure regulation. (3) Abnormalities in the RAS might lead to abnormal cholesterol function, thereby affecting blood pressure regulation.

There exists an association between renal calyceal diverticulum and hypertension; hypertension can thus be a surgical indication in cases of renal calyceal diverticulum. In addition, renal calyceal diverticulum can be easily misdiagnosed as a renal cyst in children. Hence, it is important to be vigilant to prevent a series of complications, such as postoperative urine leakage, in such cases.

## Data Availability

The datasets used and/or analysed during the current study are available from the corresponding author on reasonable request.

## Abbreviations

CT: Computed tomography
CTA: Computed tomography angiography
RAS: Renin-angiotensin system
NADH: Nicotinamide adenine dinucleotide
